# Circulating tumour cells in gastrointestinal cancers: food for thought?

**DOI:** 10.1038/s41416-023-02228-8

**Published:** 2023-03-17

**Authors:** Simran Asawa, Manuel Nüesch, Ana Gvozdenovic, Nicola Aceto

**Affiliations:** Department of Biology, Institute of Molecular Health Sciences, Swiss Federal Institute of Technology Zurich (ETH Zurich), Zurich, Switzerland

**Keywords:** Cancer, Metastasis

## Abstract

Gastrointestinal (GI) cancers account for 35% of cancer-related deaths, predominantly due to their ability to spread and generate drug-tolerant metastases. Arising from different locations in the GI system, the majority of metastatic GI malignancies colonise the liver and the lungs. In this context, circulating tumour cells (CTCs) are playing a critical role in the formation of new metastases, and their presence in the blood of patients has been correlated with a poor outcome. In addition to their prognostic utility, prospective targeting of CTCs may represent a novel, yet ambitious strategy in the fight against metastasis. A better understanding of CTC biology, mechanistic underpinnings and weaknesses may facilitate the development of previously underappreciated anti-metastasis approaches. Here, along with related clinical studies, we outline a selection of the literature describing biological features of CTCs with an impact on their metastasis forming ability in different GI cancers.

## Introduction

Gastrointestinal (GI) cancers are diagnosed in one out of four cancer patients worldwide. They comprise a group of malignant tumours originating from various locations along the GI tract, from the oesophagus to the anus, including supportive glands like liver and pancreas [1]. The most common GI cancers arise in the epithelial tissue of the oesophagus, stomach, pancreas, liver, and colorectum. With 4.9 million new cases and 3.9 million deaths in 2020, GI cancers are among the leading cause of cancer-related fatality in both genders [2].

The majority of GI cancer mortality is associated with metastasis—a complex, multistep process involving systemic spread of tumour cells from the primary site throughout the body, followed by colonisation of secondary organs. The standard of care for GI cancer patients includes approaches such as surgery, chemotherapy, radiotherapy, targeted therapy and immunotherapy. However, once the tumour has metastasised to distant organs, with lungs and liver being the most frequent sites, therapeutic options become increasingly narrower [3–6]. Typically, metastatic lesions become clinically visible in GI cancer patients in the years that follow surgical removal of the primary tumour [7–10]. Hence, identifying key drivers of the metastatic process is an essential step to facilitate the development of novel therapeutic strategies that may prevent and/or eradicate metastasis.

In case of most solid tumours, including GI malignancies, growing cancerous lesions may shed cells in the bloodstream which can travel to distant organs and form metastases. These pioneers of the metastatic process are referred to as circulating tumour cells (CTCs). Though relatively little is known about the mechanisms that influence dissemination and seeding of CTCs, it is well proven that increased numbers of CTCs in blood correlate with poor prognosis in patients [11]. Owing to the rarity of these cells in the peripheral circulation and a limited set of biomarkers for their identification, isolation of CTCs has been notoriously challenging [12]. Several technologies, each with their own set of advantages and disadvantages, are employed for CTC isolation in GI cancers. They can be broadly divided into two categories: antigen-dependent (based on the unique markers expressed by tumour cells but absent in other circulating blood cells) and antigen-independent (agnostic to markers but based on specific biophysical characteristics of CTCs that differ from haematological cells). So far, the antigen-dependent platform CellSearch [13] and the antigen-independent microfluidics technology Parsortix [14] have been approved by the Food and Drug Administration (FDA) for use in at least one cancer type. Of note, in GI cancers, at present only the CellSearch technology has been approved for monitoring CTCs in colorectal cancer (CRC) [15].

Based on the emergence of these technologies, over the past decades, substantial clinical and research efforts have been directed towards CTCs. In this review, we summarise recent insights into their biology and clinical relevance in the most prevalent GI cancers.

## Clinical relevance of CTCs in GI cancers

Considering their pivotal role in metastatic spread, CTCs are extremely important for disease progression. In clinical settings, CTCs are utilised as prognostic biomarkers in GI cancer. CTC detection levels (and respective cut-off values) in the peripheral blood differ among cancer types and depend on the isolation method used [16]. The presence of three or more CTCs per 7.5 ml of blood using CellSearch is the FDA-approved gold standard for prognosis in CRC [17], yet more recent studies with the identical isolation method demonstrate that detection of even one or two CTCs per 7.5 ml of blood is associated with unfavourable prognosis in CRC patients [18, 19]. In addition to the prognostic value of CTC, the investigation of CTC suitability for early cancer diagnosis, evaluation of therapeutic response and monitoring of recurrence after surgery is enabled by recent technological advances [20–24]. Here and in Table 1, we provided the most relevant studies investigating the clinical relevance of CTCs in the five most prevalent GI cancers in the recent years.

**Table 1:** Clinical relevance of CTCs in GI cancers (studies published between 2017 and 2022).

Cancer type	No. of patients	Detection methods	Main findings/clinical trial no.	Ref.
Oesophageal cancer	115	ISET	Preoperative chemotherapy and CTC detection prior to treatment correlates with better short-term PFS for patients with stage II or III ESCC (NCT03005314)	[122]
	96	CellSearch	The presence of CTCs in patients during follow-up after tri-modality therapy was associated with significantly poorer DFS and OS regardless of the timing of chemoradiotherapy in non-metastatic oesophageal cancer patients (NCT00907543)	[26]
Gastric cancer	116	Centrifugal microfluidic system	CTC count (≥2 cells/7.5 ml blood threshold) after gastrectomy does not correlate with gastric cancer staging. CTC potentially used as an early diagnostic marker (85.3% sensitivity and 90.3% specificity)	[27]
	162	CellSearch	Dynamic changes in CTC count (≥2 cells/7.5 ml blood threshold) between baseline and at day 28 after treatment were significantly associated with PFS and OS (NCT01443065)	[28]
	93	CellSearch	Pre and postoperative CTC levels are prognostic markers for recurrence of advanced gastric cancer after resection	[123]
	228	Flow cytometry, immunofluorescent double staining	CK^+^CD44^+^ cells were significantly more common among patients with distant metastases and were associated with significantly shortened survival	[30]
	100	Ficoll	FGFR2^+^ CTCs (≥5 cells/10 ml blood) were associated with poorer RFS. CTCs might be helpful to identify an existing tumour with FGFR2 overexpression	[31]
	70	Immunofluorescence	CSV^+^PD-L1^+^ CTCs are significantly correlated with short survival duration and poor therapeutic response	[32]
	150	Ficoll	EpCAM^−^CEA^+^ cells count was significantly associated with 3-year RFS	[33]
Hepatocellular carcinoma	62	Multiplex fluorescence in situ hybridisation	Mesenchymal-like CTCs (≥1 cells/5 ml blood threshold) are correlated with the risk of early recurrence	[124]
	85	Flow cytometry	Preoperative GPC3^+^ CTCs (≥5 cells/8 ml blood threshold) are associated with poor prognosis and are a risk factor for microscopic portal vein invasion (UMIN000025989)	[125]
	105	Tapered slit filter	Postoperative CTCs were detected in 23.8% of HCC patients and associated with lower survival and higher recurrence in early HCC	[35]
	256	Canpatrol	CTC count and EMT status were not associated with predictive recurrence and clinical stages	[23]
	197	CellSearch	Postoperative CTCs (≥3 cells/7.5 ml blood) count is predictive of postoperative extrahepatic metastases	[22]
Pancreatic cancer	60	ISET, immunofluorescence	ALDH^+^ CD133^+^ CD44^+^ CTCs are predictors of tumour recurrence and associated with decreased disease-free and OS	[43]
	46	Specific TU-chip	Phenotypic-based CTC analysis was used to predict metastasis (hybrid: epithelial and mesenchymal) and OS (epithelial CTC)	[126]
	20	CellSearch	High number of CTCs (>10 cells/6 ml blood) was associated with OS and PFS. Expression of ALCAM, POU5F1B, and SMO mRNAs in CTCs is negatively correlated with shorter PFS and OS	[127]
Colorectal cancer	66	CanPatrol, in situ hybridisation	LGR5^+^ CTCs and mesenchymal-like CTCs correlated with the occurrence of metastasis	[128]
	153	CellSearch	CTC count (≥3 cells/7.5 ml blood) at baseline correlated with shorter OS (NCT01442935)	[129]
	121	Cyttel	Advanced CRC patients with CTC detection during chemotherapy had worse PFS and OS	[130]
	667	CellMax	CTC counts demonstrated high sensitivity in detecting CRC (stages I–IV)	[131]
	44	CellSearch	Presence of CTCs (≥2 cells/7.5 ml blood) was associated with disease progression and poor survival despite complete resection	[19]
	26	Cytology-based automated CTC detection	The number of CTCs in draining venous blood (mesenteric vein) was higher than in peripheral blood and increased significantly with stage progression	[132]
	1202	CellSearch	Elevated baseline CTCs (≥3 cells/7.5 ml blood) was detected in 41% of the patients. RAS mutation correlated with poor prognosis (NCT01640405, NCT01640444)	[45]
	349	CellSearch	CTC count (≥3 cells/7.5 ml blood) at baseline correlated with poor prognosis in metastatic CRC patients and might be useful as a biomarker for intensive first-line therapy decision (NCT01640405)	[133]
	168	EPISPOT, CellSearch	CTC detection at D28 and the D0–D28 CTC dynamics (after first line of treatment with FOLFIRI–bevacizumab) was associated with PFS and OS (NCT01596790)	[134]
	20	CellSearch	CTC levels are independent prognostic markers in KRAS mutated metastatic CRC patients	[135]

This table provides an overview of studies investigating the clinical relevance of CTCs in the five most prevalent GI cancers in recent years.

*ALDH* aldehyde dehydrogenase, *CEA* carcinoembryonic antigen*,* *CK* cytokeratin, *CTC* circulating tumour cell, *CRC* colorectal cancer, *CSV* cell surface vimentin, *EMT* epithelial–mesenchymal transition, *EpCAM* epithelial cell adhesion molecule, *ESCC* oesophageal squamous cell carcinoma, *FGFR2* fibroblast growth factor receptor 2, *GPC3* glypican 3, *HCC* hepatocellular carcinoma, *LGR5* leucine-rich repeat-containing G-protein couple receptor 5, *OS* overall survival, *PDAC* pancreatic ductal adenocarcinoma, *PD-L1* programmed cell death ligand-1, *PFS* progression-free survival, *POU5F1B* putative pou domain class transcription factor 1B*,* *RFS* recurrence-free survival, *SMO* smoothened.

In metastatic oesophageal cancer (OC), prognosis based on CTC analysis has been described as promising in numerous studies but with some limitations (e.g. lack of optimal cut-off value consensus) [25]. In non-metastatic OC patients undergoing tri-modality therapy (combination of radiotherapy, chemotherapy and surgery), CTCs were interrogated as potential prognostic indicators [26]. Using the CellSearch system, the presence of CTCs 6, 12 and 24 months after treatment was associated with significantly poorer disease-free and overall survival, regardless of the timing of chemoradiotherapy (neoadjuvant vs adjuvant chemoradiation). The results of an ongoing Canadian clinical trial (NCT02812680), using a large OC patient cohort (*n* = 200) may contribute to understanding the utility of CTCs and plasma microRNA in OC cancer management.

In gastric cancer (GC), a threshold of two or more CTCs per 7.5 ml of blood was used for differentiating patients with GC from healthy controls [27]. CTCs were identified in 85% of GC patients, but no associations between CTCs and clinicopathologic features such as histologic type, T stage, N stage, and mucin phenotype were found. Interestingly, more than 80% of the patients with early-stage disease (T1, N0) had detectable CTCs, implicating their use as an early diagnostic marker [27]. CTC levels, quantified at baseline and day 28 upon chemotherapy treatment initiation, were also reported to be a prognostic factor in GC, as they were associated with worse progression-free and overall survival [28]. Of note, CTCs often appear to be heterogeneous and in rare instances, some carcinoma-derived CTCs may even reduce the expression of their epithelial markers [29]. Therefore, antigen-based isolation using a single marker (e.g EpCAM) may underestimate the phenotypic diversity of CTCs. Efforts are made to increase the variety of CTC markers to further improve their characterisation [29]. For example, CD44, cytokeratin, fibroblast growth factor, cell surface vimentin protein, programmed cell death ligand-1, carcinoembryonic antigen, and HER2-positive CTCs were used as potential prognostic markers in GC [30–34].

In hepatocellular carcinoma (HCC), CTC counts before and after surgery were compared in early-stage HCC using different isolation methods, but contradictory outcomes were reported [22, 23, 35]. This illustrates again the importance of standardised CTC-detecting assays, able to capture heterogeneous and rare CTC in various contexts. In this direction, using a 113-patient cohort, CTC enumeration and phenotypic features were interrogated using CanPatrolTM technology. This technology is based on the enrichment of CTCs by combining red blood cell lysis, depletion of CD45-positive cells via magnetic bead separation method, and subsequent size-based isolation of CTCs [36]. Levels of CTCs and their respective phenotypes were not correlated with clinical stages or predictive of recurrence in HCC.

The discovery of biomarkers that could guide treatment decisions is also crucial in pancreatic ductal adenocarcinomas (PDACs). Neoadjuvant therapy in early-stage disease could improve patient survival [37–39]. The presence of three or more CTCs per 4 ml of blood is associated with shorter recurrence-free survival following surgery as well as worse overall survival [40]. CTCs expressing vimentin, a mesenchymal cell-associated surface marker, were detected in 76% of pancreatic cancer patients [41, 42]. The detection of vimentin-positive CTCs preoperatively correlated with change in the tumour burden (more advanced disease and metastasis) and short recurrence-free survival. Using isolation by size of epithelial tumour method, mesenchymal-like or stem-like CTC were also detected in pancreatic cancer [43].

Efforts made to assess the clinical validity of CTCs in non-metastatic cancer are of interest too, particularly for the identification of early treatment opportunities. A meta-analysis including 20 studies (*n* = 3687 patients) demonstrated that CTC detection in blood (presence of one or more CTCs per 7.5 ml of blood) of patients with non-metastatic CRC was correlated with aggressive disease progression and reduced disease-free survival [44]. Postoperative CTCs correlated with poor recurrence-free survival [24]. A randomised phase III clinical trial analysed the correlation between baseline CTC, molecular profiling and clinical characteristics [45]. Elevated baseline CTCs and RAS mutations were associated with poor clinicopathologic prognostic factors, such as stage IV at diagnosis and involvement of at least three metastatic sites. Similarly, analysis of subgroups of CRCs patients (left vs right hemi-colon and colon vs rectal cancer) revealed a correlation between CTC positivity (presence of three or more CTCs per 7.5 ml of blood) and anatomical location of the tumour [21]. Furthermore, quantitative and phenotypic heterogeneity of CTCs was observed in distal compared to proximal CRCs and distinct features of CTCs in left-sided colon cancer may be accountable for poor prognosis observed within this subgroup of patients [46].

Overall, while seemingly heterogeneous, GI CTCs appear to be promising biomarkers for monitoring cancer progression. Ultimately, the clinical utility of CTCs will strongly depend on their ability to be implemented in standard clinical practice and to provide useful information to aid clinical decisions.

## Biology of CTCs in GI cancers

### Molecular features of CTCs

One of the initial steps in the metastatic cascade involves detachment of tumour cells from the primary tumour by breaching through the basement membrane and invading the adjacent tissue, which is counterintuitive given the poorly-motile, adult epithelial cell origin of carcinomas [47]. To understand how tumour cells equip themselves for this crucial step, various models have been proposed. In vitro and in vivo studies found that metastatic cells can travel individually via an epithelial-to-mesenchymal transition (EMT)—an embryonic development process of *trans*-differentiation of epithelial cells to cells with a mesenchymal-like phenotype, contributing to their ability to invade, withstand stress and disseminate [48, 49]. Recent studies have revealed that, rather than epithelial- and mesenchymal-like extremes, this process involves a spectrum of transitional phases where these two states are somewhat plastic [50, 51]. In contrast, besides travelling individually as single cells, CTCs can also travel as clusters of two or more cells. Such CTC clusters have higher metastatic seeding capability compared to single cells [52]. CTC clusters can be homotypic (consisting of only tumour cells) or heterotypic (where tumour cells are accompanied by non-tumour cells) [53, 54]. For instance, CTC–neutrophil clusters, observed in both breast cancer patients and mouse models, greatly contribute to metastasis given their high proliferative abilities [53]. In addition to heterotypic cluster formation with immune cells, heterogeneous clustering of tumour cells with cancer-associated fibroblasts (CAFs) has also been described in a lung cancer murine model, though their metastatic potential remains unclear [54]. Further, clusters of CTCs with polymorphonuclear myeloid-derived suppressor cells were found in metastatic melanoma and breast cancer patients [55]. This heterotypic interaction promotes the metastatic potency of CTCs via ROS/Notch/Nodal signalling.

In the context of GI cancer, CTCs have been reported as both single cells and multicellular aggregates. CTC clusters along with single CTCs were detected using size-based isolation in CRC patients, where their abundance and vimentin expression correlated with inferior prognosis [56]. Similarly, the presence of clusters has also been associated with worse survival in PDAC patients [57]. In GC, while the vast majority of detected CTC clusters consisted of two CTCs, CTC clusters with three-to-four cells were associated with therapeutic resistance and poor prognosis [58]. Of note, primary tumours can also shed non-cancer circulating entities such as cancer-associated macrophage-like cells in pancreatic cancer [59] and circulating non-malignant endothelial cell clusters in CRC [60]; however, their impact in disease progression remains unclear.

Though a prognostic value for CTC enumeration has been clinically proven, their in-depth molecular characterisation may lead to improved disease management and precision medicine. Isolation of CTCs without altering their transcriptome during processing has been considered a major challenge [12]. So far, most studies have performed bulk RNA sequencing or targeted sequencing analysis, while only a handful of published studies subjected CTCs to single-cell RNA sequencing (scRNA-seq) analysis, a powerful tool allowing for a higher-resolution dissection of tumour complexity. In one of the first efforts, RNA sequencing of orthotopic pancreatic cancer murine model-derived and patient-derived CTCs was performed using antigen-independent microfluidic isolation [61]. The data revealed at least three distinct CTC populations, highlighting their heterogeneity, where the majority possessed low proliferative signatures and enriched stem cell-associated genes like aldehyde dehydrogenase 1 family member a2 (*Aldh1a2*). CTCs displayed expression of both epithelial and mesenchymal markers along with high levels of insulin-like growth factor binding protein 5 (*Igfbp5)* transcript. When studied at the level of primary tumour using RNA in situ hybridisation, this extracellular growth factor binding was found to be focally expressed at the epithelial–stromal interface [61]. In HCC, patient-derived CTCs were characterised through a newly developed technology that combines image flow cytometry and single-cell mRNA sequencing [62]. In this proof-of-concept study, the authors describe differential gene expression between CTCs isolated from the same patient and across patients, as well as an enrichment of gene sets commonly associated with xenobiotic metabolism, coagulation, and peroxisomes, as expected considering their hepatic origin. Of note, this analysis was conducted using a limited number of cells obtained from two patients [62].

In a more recent study focusing on metastatic GC, gel-based cell manipulation was employed for antigen-agnostic size-based isolation of single CTCs from patients’ blood. The subsequent transcriptomic analysis of single CTCs revealed a characteristic gene expression profile, implying that a majority of these cells may have undergone an EMT [63]. Moreover, the results suggested that the EMT was induced by adhesion of CTCs to platelets within the blood vessels. Additionally, these EMT-induced cells exhibited cell cycle arrest and acquired chemoresistance. Of note, a small fraction of CTCs was epithelial and did not express any platelet-adherence associated genes. These epithelial cells were metabolically more active than other CTCs and correlated with a poor prognosis of the patients [63].

Although single-cell analysis is informative, naturally occurring low numbers of CTCs are a limitation for robust interpretation. To combat this, attempts have been made to culture CTCs and expand them ex vivo, aiming to augment the material for subsequent experimental interrogation [16]. Autologous cell lines were generated from colon CTCs, and high numbers of CTCs (~300 cells) were needed for ex vivo culture [64, 65]. Such ex vivo models can be instrumental in assessing functional properties of CTC and accelerating drug development efforts [66].

Altogether, with regard to molecular features, the scientific literature indicates various degrees of heterogeneity in GI CTCs, yet, further studies are required to gain more knowledge on different types of GI cancers and how their biology influences CTC generation dynamics.

### Metastatic patterns

The metastatic spread to distant organs does not appear to be a random process—as different cancer types preferentially metastasise to specific sites—a process referred to as organotropism [67]. Organ-specific metastasis is dictated by several factors including the anatomical location, circulation pattern, organ-specific niches, tumour intrinsic factors, and the interaction between the tumour cells and host microenvironment. Differences in the genomic makeup of metastases according to their organ location were also identified [68]. Furthermore, the vascular architecture within the GI tract contributes to the specific metastatic pattern of GI cancers. The hepatic portal system transports the blood, through the portal vein, from most of the GI tract to the liver. The liver is a densely vascularised organ, receiving dual blood supply (both from the hepatic arteries and the portal vein) and harbouring a rather fenestrated endothelial layer, favouring a permissive environment for CTC extravasation. The described vascular organisation is in line with the clinical observation depicting the liver as the most common site of metastases in GI cancers followed by peritoneal metastases [69, 70]. Occasionally however (e.g. distal rectum), the venous blood drainage bypasses the liver and directly flows into the lung via the inferior vena cava, resulting in a higher proportion of lung metastases in rectal cancer compared to colon cancer (20% vs 8%) [69]. In HCC, CTCs exit the liver through the hepatic veins, pass via the heart (via the inferior vena cava) and then reach the lungs, where most metastases are found [71].

Currently, the spatial representation of CTCs within anatomically distinct regions of the human circulatory system has been limited mostly due to detection (e.g. low abundance of CTCs) and accessibility issues (access to different blood vessel locations). Characterisation of CTCs within distinct compartments could be informative and provide an insight into their distinct phenotypes (i.e. abundance, morphology, heterogeneity), specific molecular features, as well as their intrinsic metastatic ability. Interestingly, comparing mesenteric venous blood versus central venous blood compartments, higher detection rate and abundance of CTCs was shown in 200 post-surgery CRC patients using the CellSearch system [72]. Further, transcriptional heterogeneity in CTCs was explored upon drawing the blood from four key vascular compartments (i.e. portal, hepatic, peripheral veins, and peripheral artery) in 10 HCC patients [73]. A single-cell level gene expression analysis (133 cells in total) revealed that CTCs isolated from different vascular compartments clustered by the patient of origin, indicating that interpatient heterogeneity is higher than intrapatient, intervascular compartment heterogeneity. Yet, the gene expression profiles of CTCs across the different compartments were further analysed within individual patients and highlighted interesting differences (e.g. cell cycle and immune response genes) between compartments but also within the same blood vessel. Notably, the heterogeneity of CTCs was significantly decreased in the peripheral artery compared to the other vascular compartments, suggesting a selection of CTCs that passed through the lung capillaries [73]. A higher number of sequenced CTCs will be required for further validation of these interesting observations. In pancreatic and bile duct cancer, a study collected portal vein blood from 41 patients and cultivated FACS-isolated CTCs and immune cells using a patient-derived ex vivo platform [74]. By adding portal blood mononuclear cells to the platform, they reconstituted the CTC and immune cell interactions that are characteristic of the portal venous system. After seven days in culture, CTCs and immune cells formed clusters which promoted CTC survival and growth in vitro [74].

Moreover, peritoneum dissemination is also observed in GI cancers and peritoneal carcinomatosis is considered to be the end stage of the disease. Especially in CRC, tumour cells form clusters to evade anoikis and shed into the lymphatic system, leading to peritoneal dissemination as opposed to the invasion-metastasis cascade that occurs during hematogenous dissemination [75]. CTCs may travel through lymphatic vessels but how cancers choose their route of dissemination is debated [76].

Altogether, CTC properties in GI cancers are affected by multiple factors including anatomical and biochemical features. Mechanical cues such as shear stress, size restriction and mechanical trapping may also impact CTC composition. A better understanding of these phenomena in GI cancer is likely to reveal unexpected, yet potentially druggable metastasis-relevant patterns.

### Tumour microenvironment

Until a few decades ago, cancer was thought to be exclusively a disease of abnormal tumour cells, generated through the accumulation of genetic aberrations. It is now widely appreciated that, additionally to the neoplastic cell components, microenvironmental elements such as stromal and immune cells play a pivotal role in cancer progression. In epithelial malignancies like colon and oesophageal carcinomas, tumours with more than 50% stromal composition correlate with unfavourable outcome [77].

In GI cancers, CAFs constitute a significant component in the stroma and their implication in metastasis, through various mechanisms, has been reported [78–80]. CAFs activate CXCL12/CXCR4 axis by integrin β1 clustering on the cell surface, enabling GC cell invasion [81]. Co-injection of patient-derived CAFs along with pancreatic cancer cells in mice leads to higher metastatic burden with an increasing proportion of co-injected CAFs [82]. Recent scRNA-seq studies have highlighted heterogeneity of CAFs in pancreatic cancer [83]. Along with plasticity amongst different phenotypic signatures, CAFs also change their role of pro-resistance to pro-invasion as pancreatic cancer progress through clinical stages [84]. Though studies have shown that CAFs support metastasis and interact with CTCs as described earlier, the impact of these interactions remains incompletely understood. CAFs can also engage and alter non-cellular components like the extracellular matrix (ECM) [85], and alterations in biomechanical properties such as ECM stiffness may trigger migration of cancer cells [86]. In CRC, matrix metalloproteinase-independent migration of cancer cells can be achieved by remodelling the basement membrane through CAF-induced biophysical forces [87]. Further, fragments of ECM components like collagen, laminins, elastins, and proteoglycans have been implicated as circulatory biomarkers and liaisons to metastasis [85, 88]. Molecular analysis of pancreatic CTCs revealed high expression of core matrisome ECM glycoproteins, such as SPARC, MGP and SPON2 [61]. Short-hairpin RNA-mediated SPARC knockdown in pancreatic cancer cells resulted in reduced migration in vitro as well as decreased metastatic burden in vivo following an orthotopic primary tumour-derived or tail vein injection into NSG mice [61]. Thus, ECM-related proteins impact metastasis but how they influence CTC dissemination remains unclear.

In addition to CAFs, immune cells are an impactful component in the GI tumour microenvironment (TME). Pro-tumour immune populations include M2 macrophages, N2 neutrophils, regulatory T cells and myeloid-derived suppressor cells; each contributing to the tumour aggressiveness via key effector molecules like colony stimulating factor-1, interleukin (IL)-6, metalloproteases, vascular endothelial growth factor, prostaglandin E2, transforming growth factor-*β* and IL-10 [89]. High levels of C-X-C chemokine motif ligand 5 (CXCL5) in the TME facilitate metastasis in GC by promoting invasion and migration via induction of EMT through activation of ERK signalling pathway in cancer cells [90]. Additionally, CXCL5 prompts activation of pro-tumour neutrophils via ERK and p38 signalling, resulting in elevated inflammatory cytokines like IL-6 and IL-23 that support metastatic potential of GC cells [90]. Tumour-associated macrophages are another immune cell type extensively studied for their contribution at each step of the metastatic cascade [91]. M2 polarisation of macrophages has been associated with inflammation in the TME, which in turn fosters metastatic progression of GI cancers [92]. A strong correlation between M2 and tumour neo-vessels has been described, suggesting their role in promoting angiogenesis and evolution of the tumour vasculature. Disorganised and collapsed neo-vessels accompanied by swift overgrowth of tumour cells leads to the development of hypoxic regions [93]. It has been reported that hypoxia triggers the intravasation of clustered breast CTCs highlighting the importance of the tumour biochemical landscape in the metastatic dissemination [94].

Overall, the complex interactions between the TME and cancer cells have a great impact on metastasis. However, the available evidence in GI cancer is limited to only few cell types and more comprehensive studies are required to fully understand these processes. Further exploration is needed to determine how biochemical and metabolic conditions of GI tumours influence CTC properties during the metastatic cascade.

### Microbiota’s contribution to metastasis

Accumulating evidence points to the microbiota as a new component of the TME. The GI tract concentrates complex and large microbial communities (i.e. bacteria, archaea, eukaryotes, viruses) that can regulate cancer onset, progression, metastasis, and response to therapy [95–97]. Numerous preclinical and clinical studies have revealed that particular bacterial species, among other microorganisms, may have tumour-promoting or tumour-preventing effects in various types of cancer. Microbiota can directly facilitate tumourigenesis through mutagenesis by inducing DNA damage directly, interfering with mechanisms that maintain genome integrity or by activating oncogenic signalling pathways [96]. On the other hand, the microbiota and their metabolites can prevent cancer through their interaction with the host immune system. Similarly, microbes play a dual role in the metastatic spread: some species stimulate antitumoural immunity, while others promote a pro-tumourigenic inflammation at the metastatic sites [96]. These effects are regulated via signalling through metabolites, an immunosuppressive microenvironment, EMT, and gut vascular barrier impairment [97]. An imbalance of gut’s microbial community, termed dysbiosis, causes the disruption of the mucosal barrier, which allows the microbes, mostly bacteria, to spread to other organs by entering the bloodstream or lymphatic system directly through the disrupted epithelial and vascular barriers. Bacteria can also travel from the primary tumour environment to distant organs by invading cancer or immune cells [98–100]. *Escherichia coli*, for example, migrate to the liver and contribute to the pre-metastatic niche maturation by promoting the recruitment of innate immune cells and the formation of an inflammatory pro-metastatic environment [101]. Interestingly, when comparing the composition of the microbiota of primary CRC tumours and hepatic metastasis, it was found that they are both colonised by similar bacteria, such as *Fusobacterium nucleatum*, *Bacteroides fragilis* and *Prevotella* species [102].

A link between the gut microbiome, its metabolites, and immune responses in the liver was described in one primary and three metastatic mouse models of liver cancer [103]. Mechanistically, this study demonstrated that the microbiota modifies the bile acid inducing an antitumour effect with the accumulation of CXCR6-positive natural killer T cells. Conversely, lipopolysaccharide, produced by *Escherichia coli*, promotes CRC metastasis in a syngeneic mouse model [104]. Lipopolysaccharide increases the secretion of cathepsin k, which in turn mediates M2-like macrophage polarisation and promotes metastasis. In the clinical settings, recent studies highlight the emerging role of the microbiota as a diagnostic and prognostic marker in GI cancers [105–107]. In GC, the presence of *Helicobacter pylori*, a bacterium colonising the stomach and classified as a carcinogen, is also correlated with a better prognosis, highlighting its controversial role [108, 109]. By modelling the gut microbiota, *H. pylori* is involved at early stage of gastric carcinogenesis but is also suggested to be absent in the later stages of tumourigenesis. In a recent pan-cancer study analysing 35 cancer types, a potential prognostic value of fungi was suggested [106]. The authors characterised the cancer mycobiome of 17,401 tissue and blood samples and found cancer-type specific mycobiomes. Noteworthy, gut microbiota do not only affect cancer progression but also impact responses to chemotherapy, radiation and immunotherapy, mainly due to a role in drug metabolism [110, 111]. Recently, faecal microbiota transplantation was proposed as a promising therapeutic strategy to compensate for microbiota dysbiosis and to improve antitumour immune response [112]. However, long-term consequences of modifying the composition of gut microbiota, e.g. via faecal microbiota transplantation, will need to be addressed in larger randomised studies. Microbiota-targeted treatments in cancer show different responses across tumours, possibly due to the heterogeneity of the microbiome composition between patients and cancer types [113]. In pancreatic cancer, for example, the absence of microbiota correlates with a better response to PD-1 immunotherapy in preclinical models [114]. In particular, bacterial ablation modulates the immune component of the TME, as reflected by the reduced numbers of myeloid-derived suppressor cells, an increase in M1 macrophage differentiation, as well as an increased fraction of intratumoural T cells.

Currently, little is known about the interactions between the microbiota and CTCs across various cancer types. A recent study demonstrated that the distribution of the microbiota within a tumour is organised in micro-niches and promotes cancer progression in oral squamous cell carcinoma and CRC patients [100]. Using single-cell RNA-sequencing and in situ spatial-profiling technologies, the identity and in situ location of intratumoural microbial communities within the tumour were revealed. Co-culturing of tumour-isolated bacteria and CRC spheroids within a collagen gel containing myeloid cells, resulted in the recruitment of myeloid cells to the tumour spheroids, modification of the transcriptome of CRC cells and facilitation of cancer cell migration [100]. Interestingly, a conserved intracellular bacterial profile in murine and human breast cancer was previously reported [115]. These intracellular bacteria, while not being required for primary tumour growth, promoted CTC survival by enhancing resistance to mechanical stress through reorganisation of the actin cytoskeleton. Depletion of these intracellular bacteria reduced lung metastasis in experimental animals. Using ISH, an enrichment of bacteria in CTC clusters and in lung metastases was detected when compared to single CTCs and primary tumour site, indicating a favourable role of specific bacteria in metastasis [115]. This concept might be extended to other cancers. We anticipate that in the GI field, future research may uncover further dynamics that govern the complex interaction between CTCs and the microbiota, providing mechanistic insights on whether and how microbiota influences the cancer dissemination and outgrowth at distant sites.

## Conclusions

**Fig. 1:**
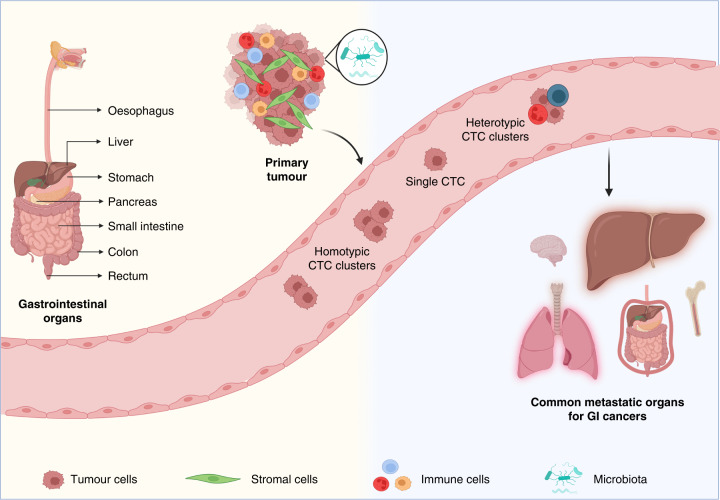
Circulating tumour cells (CTCs) in gastrointestinal (GI) cancer metastasis. Primary tumours originating in various GI organs are complex entities consisting of heterogeneous tumour cell populations entangled with tumour microenvironment (TME) components like stromal cells, immune cells and microbiota. During the process of metastasis, cancer cells intravasate into circulation as various CTC entities, including single cells, homotypic clusters and heterotypic clusters. The liver and the lungs are the predominant metastatic sites, compared to peritoneum, bones and brain. Illustrations were created with BioRender.

As extraordinarily important precursors of metastasis in various cancer types, research on the biological features of CTCs has increased over the last decades. However, compared to other solid malignancies, studies exploring GI CTCs are relatively sparse. In this review, we have focused on CTCs and their interplay with various local and systemic factors that influence metastatic behaviour in GI cancers (Fig. 1).

Future work in this area of research may involve several aspects. For instance, location of the blood draw can impact on CTCs abundance and characteristics in GI cancers (e.g. peripheral vs central locations) [72, 116, 117]. The origin and plasticity of GI cancer cells during disease progression is another important feature in the spatiotemporal dynamics of the metastatic process [118, 119]. Along these lines, a recent study identified a novel rare tumour cell population, named high-relapse cells, responsible for metastatic relapse in CRC patient samples and mouse models [120]. Further research should also take into consideration the timing of sample collection and therapeutic intervention, given recently observed effects of the circadian rhythm on CTC intravasation and metastatic ability [121]. Practically, in the case of GI cancers, both timing and location of blood collection could help capturing more (and maybe more disease-relevant) CTCs, eventually including those with higher metastatic propensity. Devices that integrate CTC capture with molecular and functional testing at specific time points may facilitate a routine application in the clinical context.

Finally, although recent research advances outlined in this review highlight the relevance of CTCs in GI cancer, several open questions remain. Owing to primary tumour heterogeneity in the GI tract, are there diverse morphological manifestations of CTCs, such as the presence of heterotypic clusters? If so, what type of non-tumour cell types partners with CTCs in GI cancers? Is the anatomical location and timing of blood draw impacting on CTC abundance and characteristics? How is the TME affecting dissemination of GI cancers? Can CTCs be exploited to improve diagnosis and treatment of GI malignancies? Answering these questions will be key to a better understanding of the metastatic process in cancers of the GI tract, and may provide prospective avenues for the development of innovative treatments.

## Data Availability

Not applicable.

## References

[1] Geesman G, Gesiotto QJ, Lalani Z, Tejani N. Anatomy of the Gastrointestinal System. In: Narayan D, Kapadia SE, Kodumudi G, Vadivelu N (eds). Surgical and perioperative management of patients with anatomic anomalies. Cham: Springer International Publishing; 2021. pp. 145–77.

[2] Sung H, Ferlay J, Siegel RL, Laversanne M, Soerjomataram I, Jemal A (2021). Global Cancer Statistics 2020: GLOBOCAN estimates of incidence and mortality worldwide for 36 cancers in 185 countries. CA Cancer J Clin.

[3] Ajani JA, Lee J, Sano T, Janjigian YY, Fan D, Song S (2017). Gastric adenocarcinoma. Nat Rev Dis Prim.

[4] Kuipers EJ, Grady WM, Lieberman D, Seufferlein T, Sung JJ, Boelens PG (2015). Colorectal cancer. Nat Rev Dis Prim.

[5] Llovet JM, Kelley RK, Villanueva A, Singal AG, Pikarsky E, Roayaie S (2021). Hepatocellular carcinoma. Nat Rev Dis Prim.

[6] Kleeff J, Korc M, Apte M, La Vecchia C, Johnson CD, Biankin AV (2016). Pancreatic cancer. Nat Rev Dis Prim.

[7] Mokadem I, Dijksterhuis WPM, van Putten M, Heuthorst L, de Vos-Geelen JM, Haj Mohammad N (2019). Recurrence after preoperative chemotherapy and surgery for gastric adenocarcinoma: a multicenter study. Gastric Cancer.

[8] Moletta L, Serafini S, Valmasoni M, Pierobon ES, Ponzoni A, Sperti C (2019). Surgery for recurrent pancreatic cancer: is it effective?. Cancers..

[9] Guraya SY (2019). Pattern, stage, and time of recurrent colorectal cancer after curative surgery. Clin Colorectal Cancer.

[10] Amin MB, Greene FL, Edge SB, Compton CC, Gershenwald JE, Brookland RK (2017). The Eighth Edition AJCC Cancer Staging Manual: continuing to build a bridge from a population-based to a more “personalized” approach to cancer staging. CA Cancer J Clin.

[11] Yang C, Chen F, Wang S, Xiong B (2019). Circulating tumor cells in gastrointestinal cancers: current status and future perspectives. Front Oncol.

[12] Radfar P, Es HA, Salomon R, Kulasinghe A, Ramalingam N, Sarafraz-Yazdi E (2022). Single-cell analysis of circulating tumour cells: enabling technologies and clinical applications. Trends Biotechnol.

[13] Riethdorf S, O’Flaherty L, Hille C, Pantel K (2018). Clinical applications of the CellSearch platform in cancer patients. Adv Drug Deliv Rev.

[14] Xu L, Mao X, Imrali A, Syed F, Mutsvangwa K, Berney D (2015). Optimization and evaluation of a novel size based circulating tumor cell isolation system. PLoS ONE.

[15] Negin BP, Cohen SJ (2010). Circulating tumor cells in colorectal cancer: past, present, and future challenges. Curr Treat Options Oncol..

[16] Eslami-S Z, Cortés-Hernández LE, Thomas F, Pantel K, Alix-Panabières C (2022). Functional analysis of circulating tumour cells: the KEY to understand the biology of the metastatic cascade. Br J Cancer.

[17] Cohen SJ, Punt CJA, Iannotti N, Saidman BH, Sabbath KD, Gabrail NY (2008). Relationship of circulating tumor cells to tumor response, progression-free survival, and overall survival in patients with metastatic colorectal cancer. J Clin Oncol.

[18] Gazzaniga P, Raimondi C, Gradilone A, Biondi Zoccai G, Nicolazzo C, Gandini O (2013). Circulating tumor cells in metastatic colorectal cancer: do we need an alternative cutoff?. J Cancer Res Clin Oncol.

[19] Arrazubi V, Mata E, Antelo ML, Tarifa A, Herrera J, Zazpe C (2019). Circulating tumor cells in patients undergoing resection of colorectal cancer liver metastases. clinical utility for long-term outcome: a prospective trial. Ann Surg Oncol.

[20] Lin D, Shen L, Luo M, Zhang K, Li J, Yang Q (2021). Circulating tumor cells: biology and clinical significance. Sig Transduct Target Ther.

[21] Pan RJ, Hong HJ, Sun J, Yu CR, Liu HS, Li PY (2021). Detection and clinical value of circulating tumor cells as an assisted prognostic marker in colorectal cancer patients. Cancer Manag Res.

[22] Sun YF, Wang PX, Cheng JW, Gong ZJ, Huang A, Zhou KQ (2020). Postoperative circulating tumor cells: An early predictor of extrahepatic metastases in patients with hepatocellular carcinoma undergoing curative surgical resection. Cancer Cytopathol.

[23] Chen Y, Li S, Li W, Yang R, Zhang X, Ye Y (2019). Circulating tumor cells undergoing EMT are poorly correlated with clinical stages or predictive of recurrence in hepatocellular carcinoma. Sci Rep.

[24] Wang D, Yang Y, Jin L, Wang J, Zhao X, Wu G (2019). Prognostic models based on postoperative circulating tumor cells can predict poor tumor recurrence-free survival in patients with stage II-III colorectal cancer. J Cancer.

[25] Li Y, Wu G, Yang W, Wang X, Duan L, Niu L (2020). Prognostic value of circulating tumor cells detected with the CellSearch system in esophageal cancer patients: a systematic review and meta-analysis. BMC Cancer.

[26] Yu E, Allan AL, Sanatani M, Lewis D, Warner A, Dar AR (2022). Circulating tumor cells detected in follow-up predict survival outcomes in tri-modality management of advanced non-metastatic esophageal cancer: a secondary analysis of the QUINTETT randomized trial. BMC Cancer.

[27] Kang HM, Kim GH, Jeon HK, Kim DH, Jeon TY, Park DY (2017). Circulating tumor cells detected by lab-on-a-disc: role in early diagnosis of gastric cancer. PLoS ONE.

[28] Pernot S, Badoual C, Terme M, Castan F, Cazes A, Bouche O (2017). Dynamic evaluation of circulating tumour cells in patients with advanced gastric and oesogastric junction adenocarcinoma: Prognostic value and early assessment of therapeutic effects. Eur J Cancer.

[29] Carneiro A, Piairo P, Teixeira A, Ferreira D, Cotton S, Rodrigues C (2022). Discriminating epithelial to mesenchymal transition phenotypes in circulating tumor cells isolated from advanced gastrointestinal cancer patients. Cells..

[30] Szczepanik A, Sierzega M, Drabik G, Pituch-Noworolska A, Kołodziejczyk P, Zembala M (2019). CD44+ cytokeratin-positive tumor cells in blood and bone marrow are associated with poor prognosis of patients with gastric cancer. Gastric Cancer.

[31] Kuroda K, Yashiro M, Miki Y, Sera T, Yamamoto Y, Sugimoto A (2020). Circulating tumor cells with FGFR2 expression might be useful to identify patients with existing FGFR2-overexpressing tumor. Cancer Sci.

[32] Liu M, Wang R, Sun X, Liu Y, Wang Z, Yan J (2020). Prognostic significance of PD‐L1 expression on cell‐surface vimentin‐positive circulating tumor cells in gastric cancer patients. Mol Oncol.

[33] Miki Y, Yashiro M, Kuroda K, Okuno T, Togano S, Masuda G (2021). Circulating CEA-positive and EpCAM-negative tumor cells might be a predictive biomarker for recurrence in patients with gastric cancer. Cancer Med.

[34] Matsushita D, Uenosono Y, Arigami T, Yanagita S, Okubo K, Kijima T (2021). Clinical significance of circulating tumor cells in the response to trastuzumab for HER2-negative metastatic gastric cancer. Cancer Chemother Pharmacol.

[35] Ha Y, Kim TH, Shim JE, Yoon S, Jun MJ, Cho YH (2019). Circulating tumor cells are associated with poor outcomes in early-stage hepatocellular carcinoma: a prospective study. Hepatol Int.

[36] Cheng Y, Luo L, Zhang J, Zhou M, Tang Y, He G (2019). Diagnostic value of different phenotype circulating tumor cells in hepatocellular carcinoma. J Gastrointest Surg.

[37] Gillen S, Schuster T, Meyer Zum Büschenfelde C, Friess H, Kleeff J (2010). Preoperative/neoadjuvant therapy in pancreatic cancer: a systematic review and meta-analysis of response and resection percentages. PLoS Med.

[38] Mokdad AA, Minter RM, Zhu H, Augustine MM, Porembka MR, Wang SC (2017). Neoadjuvant therapy followed by resection versus upfront resection for resectable pancreatic cancer: a propensity score matched analysis. J Clin Oncol.

[39] Brown ZJ, Heh V, Labiner HE, Brock GN, Ejaz A, Dillhoff M, et al. Surgical resection rates after neoadjuvant therapy for localized pancreatic ductal adenocarcinoma: meta-analysis. Br J Surg. 2022;110:34–42. 10.1093/bjs/znac354 36346716

[40] Court CM, Ankeny JS, Sho S, Winograd P, Hou S, Song M (2018). Circulating tumor cells predict occult metastatic disease and prognosis in pancreatic cancer. Ann Surg Oncol.

[41] Wei T, Zhang X, Zhang Q, Yang J, Chen Q, Wang J (2019). Vimentin-positive circulating tumor cells as a biomarker for diagnosis and treatment monitoring in patients with pancreatic cancer. Cancer Lett.

[42] Zhao XH, Wang ZR, Chen CL, Di L, Bi ZF, Li ZH (2019). Molecular detection of epithelial-mesenchymal transition markers in circulating tumor cells from pancreatic cancer patients: potential role in clinical practice. World J Gastroenterol.

[43] Poruk KE, Blackford AL, Weiss MJ, Cameron JL, He J, Goggins M (2017). Circulating tumor cells expressing markers of tumor-initiating cells predict poor survival and cancer recurrence in patients with pancreatic ductal adenocarcinoma. Clin Cancer Res.

[44] Lu Y-J, Wang P, Peng J, Wang X, Zhu Y-W, Shen N (2017). Meta-analysis reveals the prognostic value of circulating tumour cells detected in the peripheral blood in patients with non-metastatic colorectal cancer. Sci Rep.

[45] Sastre J, de la Orden V, Martínez A, Bando I, Balbín M, Bellosillo B (2020). Association between baseline circulating tumor cells, molecular tumor profiling, and clinical characteristics in a large cohort of chemo-naïve metastatic colorectal cancer patients prospectively collected. Clin Colorectal Cancer.

[46] Nicolazzo C, Raimondi C, Gradilone A, Emiliani A, Zeuner A, Francescangeli F (2019). Circulating tumor cells in right- and left-sided colorectal cancer. Cancers..

[47] Cheung KJ, Ewald AJ (2016). A collective route to metastasis: seeding by tumor cell clusters. Science..

[48] Cano A, Pérez-Moreno MA, Rodrigo I, Locascio A, Blanco MJ, del Barrio MG (2000). The transcription factor Snail controls epithelial–mesenchymal transitions by repressing E-cadherin expression. Nat Cell Biol.

[49] Takeichi M (1995). Morphogenetic roles of classic cadherins. Curr Opin Cell Biol.

[50] Fares J, Fares MY, Khachfe HH, Salhab HA, Fares Y (2020). Molecular principles of metastasis: a hallmark of cancer revisited. Sig Transduct Target Ther.

[51] Nieto MA, Huang RYJ, Jackson RA, Thiery JP (2016). EMT: 2016. Cell..

[52] Aceto N, Bardia A, Miyamoto DT, Donaldson MC, Wittner BS, Spencer JA (2014). Circulating tumor cell clusters are oligoclonal precursors of breast cancer metastasis. Cell..

[53] Szczerba BM, Castro-Giner F, Vetter M, Krol I, Gkountela S, Landin J (2019). Neutrophils escort circulating tumour cells to enable cell cycle progression. Nature..

[54] Duda DG, Duyverman AMMJ, Kohno M, Snuderl M, Steller EJA, Fukumura D (2010). Malignant cells facilitate lung metastasis by bringing their own soil. Proc Natl Acad Sci USA.

[55] Sprouse ML, Welte T, Boral D, Liu HN, Yin W, Vishnoi M (2019). PMN-MDSCs enhance CTC metastatic properties through reciprocal interactions via ROS/Notch/Nodal signaling. Int J Mol Sci.

[56] Zhang D, Zhao L, Zhou P, Ma H, Huang F, Jin M (2017). Circulating tumor microemboli (CTM) and vimentin+ circulating tumor cells (CTCs) detected by a size-based platform predict worse prognosis in advanced colorectal cancer patients during chemotherapy. Cancer Cell Int.

[57] Chang MC, Chang YT, Chen JY, Jeng YM, Yang CY, Tien YW (2016). Clinical significance of circulating tumor microemboli as a prognostic marker in patients with pancreatic ductal adenocarcinoma. Clin Chem.

[58] Chen Y, Yuan J, Li Y, Li X, Yang Y, Li J (2021). Profiling heterogenous sizes of circulating tumor microemboli to track therapeutic resistance and prognosis in advanced gastric cancer. Hum Cell.

[59] Gardner KP, Aldakkak M, Tang CM, Tsai S, Adams DL (2021). Circulating stromal cells in resectable pancreatic cancer correlates to pathological stage and predicts for poor clinical outcomes. npj Precis Oncol.

[60] Cima I, Kong SL, Sengupta D, Tan IB, Phyo WM, Lee D (2016). Tumor-derived circulating endothelial cell clusters in colorectal cancer. Sci Transl Med.

[61] Ting DT, Wittner BS, Ligorio M, Vincent Jordan N, Shah AM, Miyamoto DT (2014). Single-cell RNA sequencing identifies extracellular matrix gene expression by pancreatic circulating tumor cells. Cell Rep.

[62] D’Avola D, Villacorta-Martin C, Martins-Filho SN, Craig A, Labgaa I, von Felden J (2018). High-density single cell mRNA sequencing to characterize circulating tumor cells in hepatocellular carcinoma. Sci Rep.

[63] Negishi R, Yamakawa H, Kobayashi T, Horikawa M, Shimoyama T, Koizumi F (2022). Transcriptomic profiling of single circulating tumor cells provides insight into human metastatic gastric cancer. Commun Biol.

[64] Soler A, Cayrefourcq L, Mazard T, Babayan A, Lamy PJ, Assou S (2018). Autologous cell lines from circulating colon cancer cells captured from sequential liquid biopsies as model to study therapy-driven tumor changes. Sci Rep.

[65] Cayrefourcq L, Mazard T, Joosse S, Solassol J, Ramos J, Assenat E (2015). Establishment and characterization of a cell line from human circulating colon cancer cells. Cancer Res.

[66] Diamantopoulou Z, Castro-Giner F, Aceto N (2020). Circulating tumor cells: ready for translation?. J Exp Med.

[67] Gao Y, Bado I, Wang H, Zhang W, Rosen JM, Zhang XHF (2019). Metastasis organotropism: redefining the congenial soil. Dev Cell.

[68] Nguyen B, Fong C, Luthra A, Smith SA, DiNatale RG, Nandakumar S (2022). Genomic characterization of metastatic patterns from prospective clinical sequencing of 25,000 patients. Cell..

[69] Riihimaki M, Hemminki A, Sundquist J, Hemminki K (2016). Patterns of metastasis in colon and rectal cancer. Sci Rep.

[70] Tsilimigras DI, Brodt P, Clavien PA, Muschel RJ, D’Angelica MI, Endo I (2021). Liver metastases. Nat Rev Dis Prim.

[71] Wu W, He X, Andayani D, Yang L, Ye J, Li Y (2017). Pattern of distant extrahepatic metastases in primary liver cancer: a SEER based study. J Cancer.

[72] Rahbari NN, Bork U, Kircher A, Nimitz T, Schölch S, Kahlert C (2012). Compartmental differences of circulating tumor cells in colorectal cancer. Ann Surg Oncol.

[73] Sun YF, Wu L, Liu SP, Jiang MM, Hu B, Zhou KQ (2021). Dissecting spatial heterogeneity and the immune-evasion mechanism of CTCs by single-cell RNA-seq in hepatocellular carcinoma. Nat Commun.

[74] Arnoletti JP, Fanaian N, Reza J, Sause R, Almodovar AJ, Srivastava M (2018). Pancreatic and bile duct cancer circulating tumor cells (CTC) form immune-resistant multi-cell type clusters in the portal venous circulation. Cancer Biol Ther.

[75] Pretzsch E, Bösch F, Neumann J, Ganschow P, Bazhin A, Guba M (2019). Mechanisms of metastasis in colorectal cancer and metastatic organotropism: hematogenous versus peritoneal spread. J Oncol.

[76] Mohammed SI, Torres-Luquis O, Walls E, Lloyd F (2019). Lymph-circulating tumor cells show distinct properties to blood-circulating tumor cells and are efficient metastatic precursors. Mol Oncol.

[77] van Pelt GW, Kjær-Frifeldt S, van Krieken JHJM, Al Dieri R, Morreau H, Tollenaar RAEM (2018). Scoring the tumor-stroma ratio in colon cancer: procedure and recommendations. Virchows Arch.

[78] Kobayashi H, Enomoto A, Woods SL, Burt AD, Takahashi M, Worthley DL (2019). Cancer-associated fibroblasts in gastrointestinal cancer. Nat Rev Gastroenterol Hepatol.

[79] Sun H, Wang X, Wang X, Xu M, Sheng W (2022). The role of cancer-associated fibroblasts in tumorigenesis of gastric cancer. Cell Death Dis.

[80] Zhang T, Ren Y, Yang P, Wang J, Zhou H (2022). Cancer-associated fibroblasts in pancreatic ductal adenocarcinoma. Cell Death Dis.

[81] Izumi D, Ishimoto T, Miyake K, Sugihara H, Eto K, Sawayama H (2016). CXCL12/CXCR4 activation by cancer-associated fibroblasts promotes integrin β1 clustering and invasiveness in gastric cancer. Int J Cancer.

[82] Ligorio M, Sil S, Malagon-Lopez J, Nieman LT, Misale S, Di Pilato M (2019). Stromal microenvironment shapes the intratumoral architecture of pancreatic cancer. Cell..

[83] Lavie D, Ben-Shmuel A, Erez N, Scherz-Shouval R (2022). Cancer-associated fibroblasts in the single-cell era. Nat Cancer.

[84] Liu S, Suhail Y, Novin A, Perpetua L, Kshitiz (2022). Metastatic transition of pancreatic ductal cell adenocarcinoma is accompanied by the emergence of pro-invasive cancer-associated fibroblasts. Cancers..

[85] Brassart-Pasco S, Brézillon S, Brassart B, Ramont L, Oudart JB, Monboisse JC (2020). Tumor microenvironment: extracellular matrix alterations influence tumor progression. Front Oncol.

[86] Paolillo M, Schinelli S (2019). Extracellular matrix alterations in metastatic processes. Int J Mol Sci.

[87] Glentis A, Oertle P, Mariani P, Chikina A, El Marjou F, Attieh Y (2017). Cancer-associated fibroblasts induce metalloprotease-independent cancer cell invasion of the basement membrane. Nat Commun.

[88] Vasilaki D, Bakopoulou A, Tsouknidas A, Johnstone E, Michalakis K (2021). Biophysical interactions between components of the tumor microenvironment promote metastasis. Biophys Rev.

[89] Chimal-Ramírez GK, Espinoza-Sánchez NA, Fuentes-Pananá EM (2013). Protumor activities of the immune response: insights in the mechanisms of immunological shift, oncotraining, and oncopromotion. J Oncol.

[90] Mao Z, Zhang J, Shi Y, Li W, Shi H, Ji R (2020). CXCL5 promotes gastric cancer metastasis by inducing epithelial-mesenchymal transition and activating neutrophils. Oncogenesis..

[91] Lin Y, Xu J, Lan H (2019). Tumor-associated macrophages in tumor metastasis: biological roles and clinical therapeutic applications. J Hematol Oncol.

[92] Güç E, Pollard JW (2021). Redefining macrophage and neutrophil biology in the metastatic cascade. Immunity..

[93] Liu J, Geng X, Hou J, Wu G (2021). New insights into M1/M2 macrophages: key modulators in cancer progression. Cancer Cell Int.

[94] Donato C, Kunz L, Castro-Giner F, Paasinen-Sohns A, Strittmatter K, Szczerba BM (2020). Hypoxia triggers the intravasation of clustered circulating tumor cells. Cell Rep.

[95] Ivleva EA, Grivennikov SI (2022). Microbiota-driven mechanisms at different stages of cancer development. Neoplasia..

[96] Hanahan D (2022). Hallmarks of cancer: new dimensions. Cancer Discov.

[97] Patel M, McAllister M, Nagaraju R, Badran SSFA, Edwards J, McBain AJ (2022). The intestinal microbiota in colorectal cancer metastasis - passive observer or key player?. Crit Rev Oncol Hematol.

[98] Nejman D, Livyatan I, Fuks G, Gavert N, Zwang Y, Geller LT (2020). The human tumor microbiome is composed of tumor type-specific intracellular bacteria. Science..

[99] Diehl GE, Longman RS, Zhang JX, Breart B, Galan C, Cuesta A (2013). Microbiota restricts trafficking of bacteria to mesenteric lymph nodes by CX3CR1hi cells. Nature..

[100] Galeano Niño JL, Wu H, LaCourse KD, Kempchinsky AG, Baryiames A, Barber B (2022). Effect of the intratumoral microbiota on spatial and cellular heterogeneity in cancer. Nature..

[101] Bertocchi A, Carloni S, Ravenda PS, Bertalot G, Spadoni I, Lo Cascio A (2021). Gut vascular barrier impairment leads to intestinal bacteria dissemination and colorectal cancer metastasis to liver. Cancer Cell.

[102] Bullman S, Pedamallu CS, Sicinska E, Clancy TE, Zhang X, Cai D (2017). Analysis of Fusobacterium persistence and antibiotic response in colorectal cancer. Science.

[103] Ma C, Han M, Heinrich B, Fu Q, Zhang Q, Sandhu M (2018). Gut microbiome-mediated bile acid metabolism regulates liver cancer via NKT cells. Science..

[104] Li R, Zhou R, Wang H, Li W, Pan M, Yao X (2019). Gut microbiota-stimulated cathepsin K secretion mediates TLR4-dependent M2 macrophage polarization and promotes tumor metastasis in colorectal cancer. Cell Death Differ.

[105] Liu NN, Jiao N, Tan JC, Wang Z, Wu D, Wang AJ (2022). Multi-kingdom microbiota analyses identify bacterial–fungal interactions and biomarkers of colorectal cancer across cohorts. Nat Microbiol.

[106] Narunsky-Haziza L, Sepich-Poore GD, Livyatan I, Asraf O, Martino C, Nejman D (2022). Pan-cancer analyses reveal cancer-type-specific fungal ecologies and bacteriome interactions. Cell..

[107] Yamamura K, Baba Y, Nakagawa S, Mima K, Miyake K, Nakamura K (2016). Human microbiome *Fusobacterium nucleatum* in esophageal cancer tissue is associated with prognosis. Clin Cancer Res.

[108] Jia Z, Zheng M, Jiang J, Cao D, Wu Y, Zhang Y (2022). Positive *H. pylori* status predicts better prognosis of non-cardiac gastric cancer patients: results from cohort study and meta-analysis. BMC Cancer.

[109] Fakharian F, Asgari B, Nabavi-Rad A, Sadeghi A, Soleimani N, Yadegar A (2022). The interplay between Helicobacter pylori and the gut microbiota: an emerging driver influencing the immune system homeostasis and gastric carcinogenesis. Front Cell Infect Microbiol.

[110] Fernandes MR, Aggarwal P, Costa RGF, Cole AM, Trinchieri G. Targeting the gut microbiota for cancer therapy. Nat Rev Cancer. 2022;22:703–22. 10.1038/s41568-022-00513-x 36253536

[111] LaCourse KD, Zepeda-Rivera M, Kempchinsky AG, Baryiames A, Minot SS, Johnston CD (2022). The cancer chemotherapeutic 5-fluorouracil is a potent *Fusobacterium nucleatum* inhibitor and its activity is modified by intratumoral microbiota. Cell Rep.

[112] Chen D, Wu J, Jin D, Wang B, Cao H (2019). Fecal microbiota transplantation in cancer management: current status and perspectives. Int J Cancer.

[113] Smet A, Kupcinskas J, Link A, Hold GL, Bornschein J (2022). The role of microbiota in gastrointestinal cancer and cancer treatment: chance or curse?. Cell Mol Gastroenterol Hepatol.

[114] Pushalkar S, Hundeyin M, Daley D, Zambirinis CP, Kurz E, Mishra A (2018). The pancreatic cancer microbiome promotes oncogenesis by induction of innate and adaptive immune suppression. Cancer Discov.

[115] Fu A, Yao B, Dong T, Chen Y, Yao J, Liu Y (2022). Tumor-resident intracellular microbiota promotes metastatic colonization in breast cancer. Cell.

[116] Yang X, Bi X, Liu F, Huang J, Zhang Z (2022). Predictive efficacy of circulating tumor cells in first drainage vein blood from patients with colorectal cancer liver metastasis. Cancer Investig.

[117] Dong X, Ma Y, Zhao X, Tian X, Sun Y, Yang Y (2020). Spatial heterogeneity in epithelial to mesenchymal transition properties of circulating tumor cells associated with distant recurrence in pancreatic cancer patients. Ann Transl Med.

[118] Fumagalli A, Oost KC, Kester L, Morgner J, Bornes L, Bruens L (2020). Plasticity of Lgr5-negative cancer cells drives metastasis in colorectal cancer. Cell Stem Cell.

[119] Ganesh K, Basnet H, Kaygusuz Y, Laughney AM, He L, Sharma R (2020). L1CAM defines the regenerative origin of metastasis-initiating cells in colorectal cancer. Nat Cancer.

[120] Cañellas-Socias A, Cortina C, Hernando-Momblona X, Palomo-Ponce S, Mulholland EJ, Turon G, et al. Metastatic recurrence in colorectal cancer arises from residual EMP1+ cells. Nature. 2022;611:603–13. 10.1038/s41586-022-05402-9 PMC7616986 36352230

[121] Diamantopoulou Z, Castro-Giner F, Schwab FD, Foerster C, Saini M, Budinjas S (2022). The metastatic spread of breast cancer accelerates during sleep. Nature..

[122] Zhao Y, Han L, Zhang W, Shan L, Wang Y, Song P (2020). Preoperative chemotherapy compared with postoperative adjuvant chemotherapy for squamous cell carcinoma of the thoracic oesophagus with the detection of circulating tumour cells randomized controlled trial. Int J Surg.

[123] Zhang Q, Shan F, Li Z, Gao J, Li Y, Shen L (2018). A prospective study on the changes and clinical significance of pre-operative and post-operative circulating tumor cells in resectable gastric cancer. J Transl Med.

[124] Wang Z, Luo L, Cheng Y, He G, Peng B, Gao Y (2018). Correlation between postoperative early recurrence of hepatocellular carcinoma and mesenchymal circulating tumor cells in peripheral blood. J Gastrointest Surg.

[125] Hamaoka M, Kobayashi T, Tanaka Y, Mashima H, Ohdan H (2019). Clinical significance of glypican-3-positive circulating tumor cells of hepatocellular carcinoma patients: a prospective study. PLoS ONE.

[126] Sun Y, Wu G, Cheng KS, Chen A, Neoh KH, Chen S (2019). CTC phenotyping for a preoperative assessment of tumor metastasis and overall survival of pancreatic ductal adenocarcinoma patients. EBioMedicine..

[127] Amantini C, Morelli MB, Nabissi M, Piva F, Marinelli O, Maggi F (2019). Expression profiling of circulating tumor cells in pancreatic ductal adenocarcinoma patients: biomarkers predicting overall survival. Front Oncol.

[128] Wang W, Wan L, Wu S, Yang J, Zhou Y, Liu F (2018). Mesenchymal marker and LGR5 expression levels in circulating tumor cells correlate with colorectal cancer prognosis. Cell Oncol.

[129] Bidard FC, Kiavue N, Ychou M, Cabel L, Stern MH, Madic J (2019). Circulating tumor cells and circulating tumor DNA detection in potentially resectable metastatic colorectal cancer: a prospective ancillary study to the unicancer Prodige-14 trial. Cells..

[130] Wang L, Zhou S, Zhang W, Wang J, Wang M, Hu X (2019). Circulating tumor cells as an independent prognostic factor in advanced colorectal cancer: a retrospective study in 121 patients. Int J Colorectal Dis.

[131] Tsai WS, You JF, Hung HY, Hsieh PS, Hsieh B, Lenz HJ (2019). Novel circulating tumor cell assay for detection of colorectal adenomas and cancer. Clin Transl Gastroenterol.

[132] Tsutsuyama M, Nakanishi H, Yoshimura M, Oshiro T, Kinoshita T, Komori K (2019). Detection of circulating tumor cells in drainage venous blood from colorectal cancer patients using a new filtration and cytology-based automated platform. PLoS ONE.

[133] Aranda E, Viéitez JM, Gómez-España A, Gil Calle S, Salud-Salvia A, Graña B (2020). FOLFOXIRI plus bevacizumab versus FOLFOX plus bevacizumab for patients with metastatic colorectal cancer and ≥3 circulating tumour cells: the randomised phase III VISNÚ-1 trial. ESMO Open.

[134] Mazard T, Cayrefourcq L, Perriard F, Senellart H, Linot B, de la Fouchardière C (2021). Clinical relevance of viable circulating tumor cells in patients with metastatic colorectal cancer: the COLOSPOT prospective study. Cancers.

[135] Salvianti F, Gelmini S, Mancini I, Pazzagli M, Pillozzi S, Giommoni E (2021). Circulating tumour cells and cell-free DNA as a prognostic factor in metastatic colorectal cancer: the OMITERC prospective study. Br J Cancer.

